# Case Report: Homozygous mutation in the *ACAD9* gene revealed in a pediatric patient initially diagnosed with familial cardiac hypertrophy

**DOI:** 10.3389/fmed.2026.1824617

**Published:** 2026-07-20

**Authors:** Natalia Kotlukova, Anastasya Kadykova, Taisia Gorelova, Lana Dzik, Igor Islanov, Elena Zaklyazminskaya

**Affiliations:** 1 Children’s City Clinical Hospital Named After Z.A. Bashlyaeva, Moscow, Russia; 2 Russian Scientific Center of Surgery Named After Academician B.V. Petrovsky, Moscow, Russia; 3 North-West State Medical University Named After I.I. Mechnikov, St. Petersburg, Russia; 4 Research Center for Medical Genetics Named After Academician N.P. Bochkov, Moscow, Russia

**Keywords:** ACAD9, cardiac hypertrophy, family planning, hypertrophic cardiomyopathy, metabolic cardiomyopathy

## Abstract

**Background:**

Severe hypertrophic cardiomyopathy presenting in infancy occurs at an estimated frequency of 1:47,000 neonates and is characterized by rapid progression and poor prognosis. Differential diagnosis and timely identification of potentially treatable conditions amenable to genotype-specific therapeutic approaches are of critical importance; however, this is not always achievable in a timely manner. ACAD9 deficiency is a rare autosomal recessive disorder of mitochondrial complex I assembly and fatty acid ß-oxidation, typically presenting in early infancy with progressive cardiac hypertrophy and lactic acidosis. Differential diagnosis might be challenging due to phenotypic overlap with sarcomeric and other metabolic cardiomyopathies and limited availability of rapid diagnostic approaches.

**Methods:**

We performed comprehensive clinical, instrumental, and laboratory assessment of a 4-month-old girl with progressive biventricular hypertrophy, metabolic acidosis, persistent hyperlactatemia, and elevated NT-proBNP. Whole-exome sequencing (Illumina NovaSeq 6000; SureSelect All Exon V7) was followed by ACMG-based variant interpretation, database review (ClinVar), and segregation analysis with Sanger sequencing in the proband and parents.

**Results:**

A homozygous missense variant NM_014049.5:c.659C > T (p.Ala220Val) in the *ACAD9* gene identified and classified as likely pathogenic (LP, Class IV). Carrier status was confirmed in both consanguine parents by Sanger sequencing. The variant is located near the catalytic core of ACAD9 and has been previously associated with severe complex I impairment and riboflavin-nonresponsive disease. Despite supportive metabolic and cardioprotective therapy, the proband died from an acute metabolic crisis and progressive heart failure before the genetic testing was complete. Parents were informed about genetic results and importance of genetic counseling and testing for further family planning.

**Conclusion:**

This case highlights the clinical significance and importance of precise diagnostics in families burdened with early sibling death. Neonatal cardiomyopathy is a critical «red flag» constellation warranting urgent molecular workup and appropriate therapy adjustment. Establishing a precise molecular diagnosis—even when it cannot alter the proband’s outcome—carries essential value for family counseling, recurrence risk assessment (25% per pregnancy), and access to reproductive options including prenatal diagnosis and preimplantation genetic testing.

## 1 Introduction

Cardiac hypertrophy in infants during the first year of life poses substantial diagnostic challenges owing to the heterogeneity of etiological factors, variability of clinical manifestations, and the potential for rapid disease progression (1). Left ventricular hypertrophy accounts for approximately 25–40% of all pediatric cardiomyopathies, with the highest incidence recorded in children under 1 year of age. The reported annual incidence of infantile hypertrophic cardiomyopathy (HCM) ranges from 0.51 to 3.2 per 100,000, underscoring its epidemiological significance in this age group (2). Neonatal forms of HCM, which may manifest immediately after birth with an estimated prevalence of approximately 1 in 47,000 live births, are characterized by a rapidly progressive clinical course and carry a particularly severe prognosis. In early infancy, LVH may be accompanied by muscular hypotonia, impaired myocardial contractility, metabolic acidosis, and signs of heart failure, necessitating a broad differential diagnostic workup (3, 4).

Inherited metabolic disorders occupy an important position among the recognized causes of infantile LVH, including defects of fatty acid oxidation, mitochondrial dysfunction, and combined disorders of cellular energy metabolism (5). Mitochondrial β-oxidation defects rank among the most clinically severe metabolic conditions associated with fatal outcomes in the first months of life, and infants with these conditions may present with hypoglycemia, lactic acidosis, and progressive myocardial involvement requiring timely identification and molecular confirmation (3, 6).

One such condition is ACAD9 deficiency (OMIM:611126), caused by pathogenic variants in the *ACAD9* gene encoding an enzyme involved in both long-chain fatty acid β-oxidation and mitochondrial respiratory chain complex I assembly (3, 5). Loss of ACAD9 function leads to cellular energy deficit with preferential involvement of high-metabolic-demand organs, particularly the heart (3). Although rare, ACAD9 deficiency is a clinically significant genetic cause of early-onset cardiomyopathy, and approximately 70 cases have been reported to date, with cardiac hypertrophy representing the predominant and most life-threatening manifestation (3, 6).

Establishing a precise molecular diagnosis is critically important not only for prognosis and therapeutic decision-making, but also for genetic counseling and reproductive planning.

The aim of this case report is to present a rare observation of metabolic cardiomyopathy in an infant from a consanguineous family, confirmed by molecular genetic testing, and to highlight the practical value of diagnosis verification for both proband treatment and family counseling.

## 2 Case presentation

**FIGURE 1:**
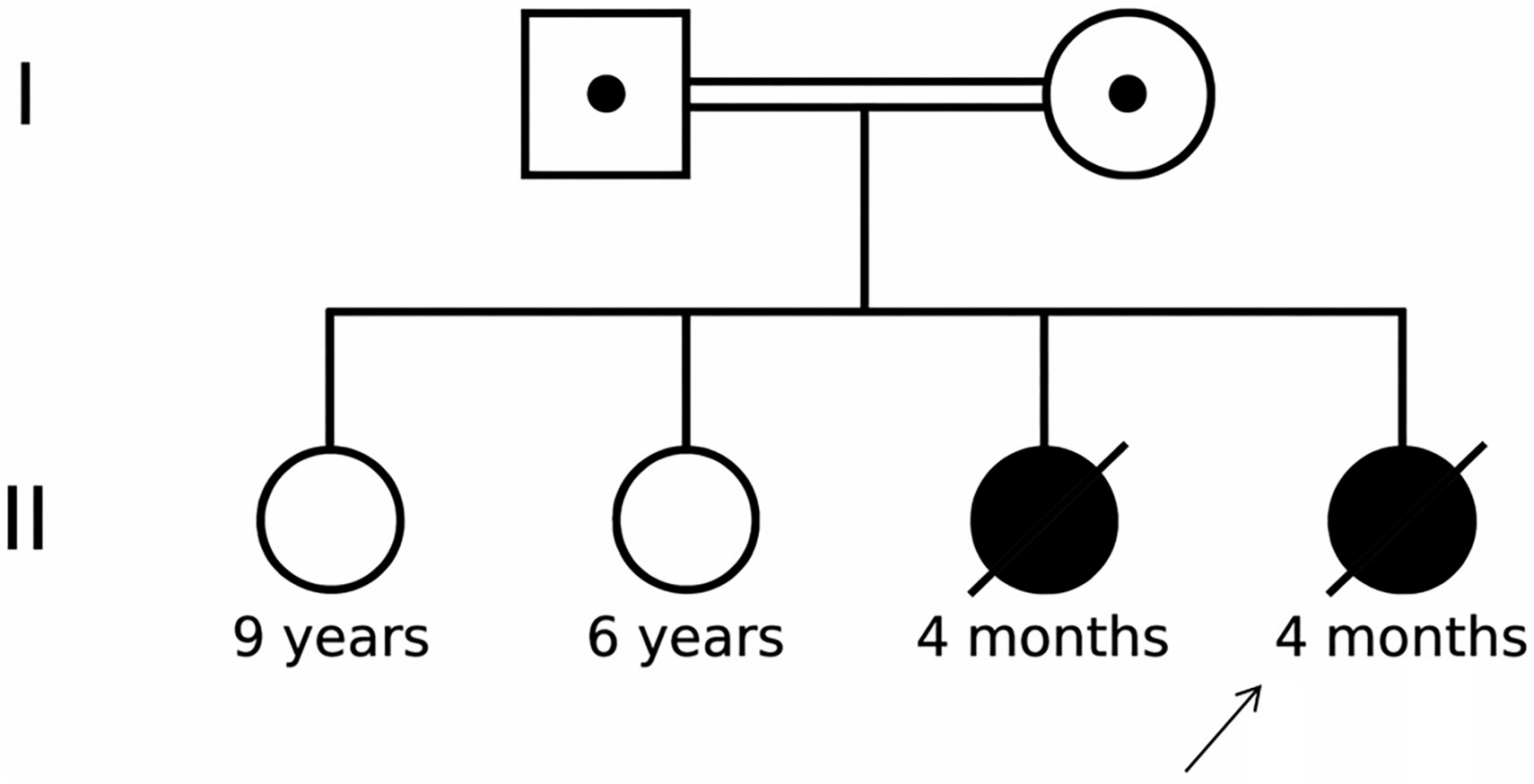
Pedigree of the family. Proband was marked by arrow.

The proband was a 4-month-old girl born to consanguineous parents (first cousins). The current pregnancy was the fourth and proceeded without significant complications. Delivery was by elective cesarean section at full term. Birth weight was 3,170 g; Apgar scores were 8/8. The family includes two healthy older sisters aged 9 and 6 years. The third pregnancy resulted in the birth of a girl who died at 4 months of age with a clinically similar presentation, presumably due to progressive cardiomyopathy (Figure 1).

From birth, the parents reported no concerns regarding the infant’s health. At 2 months of age, echocardiography was performed and revealed no pathological myocardial changes. At 3 months of age, given the significant family history, the examination was repeated, and signs of HCM were identified for the first time and coarctation of the aorta was also considered as a differential diagnosis. As the proband resided in a remote region of the Russian Federation, she was referred for comprehensive cardiological evaluation to a Federal specialized center—the Z.A. Bashlyaeva Children’s City Clinical Hospital of the Moscow Department of Health.

Following exclusion of structural cardiac defects, the patient presented with signs of catarrhal infection (rhinitis, cough) and was admitted to the infectious diseases unit with a diagnosis of acute respiratory viral infection and acute bronchitis (ICD-10: J06.9). On admission, the general condition was assessed as moderate severity. Anthropometric parameters were as follows: body weight 5,570 g, body length 62 cm, BMI 14.49 kg/m^2^. Physical examination revealed pallor, perioral cyanosis on exertion, reduced muscle tone, dyspnea, and tachycardia. Auscultation demonstrated conducted pulmonary rales with no cardiac murmurs. Hepatosplenomegaly was noted, with the liver palpable 3 cm below the costal margin and the spleen 2 cm below. Oxygen saturation (SpO2) was 97% on room air. Cytomegalovirus (CMV) infection was confirmed: CMV DNA was detected in blood, saliva, and urine, and specific antibodies were identified in peripheral blood (Table 1).

**TABLE 1:** Cytomegalovirus antibody levels at admission to the infectious diseases unit.

Parameter	Result	Units	Reference range
Anti-cytomegalovirus IgM antibodies	1.34	AI	Negative < 0.85/Positive ≥ 1.0
Anti-cytomegalovirus IgG antibodies	29.70	IU/mL	Negative < 6/Positive ≥ 6
IgG avidity index for cytomegalovirus	62.80	%	>60.00

AI, Absorbance Index (used for qualitative IgM interpretation); IU/mL, International Units per milliliter, Avidity Index > 60% indicates high-avidity antibodies, consistent with past/resolved primary infection.

Over a 20-day period, comprehensive therapy was administered, encompassing treatment of bronchitis and CMV infection, detoxification, correction of electrolyte balance and other metabolic disturbances (Table 2).

**TABLE 2:** Pharmacological therapy administered in the infectious diseases unit.

Drug	Dose	Weight-based calculation	Route of administration	Frequency	Duration
Antiviral therapy					
Ganciclovir	25 mg/dose in 40 mL NaCl	10 mg/kg/day	IV infusion pump, 40 mL/h (1 h)	Twice daily (morning, evening)	20 days
Interferon alfa-2b	150,000 IU	–	Rectal	Twice daily (morning, evening)	10 days
Gastroprotective therapy					
Omeprazole	5 mg in 50 mL NaCl	1 mg/kg/day	IV drip infusion, 50 mL/h (1 h)	Once daily (daytime)	5 days
Infusion therapy—correction of metabolic disturbances					
Dextrose 200 mL + Potassium chloride + NaHCO3 + NaCl 100 mL	300 mL single infusion	56.6 mL/kg/day	IV drip infusion	Single administration	1 day
Symptomatic therapy					
Oxymetazoline	0.2 mL	–	Intranasal	Three times daily	4 days
NaCl (normal saline)	3 drops	–	Intranasal	Three times daily	5 days

On admission, a borderline-low blood glucose level was recorded (3.8 mmol/L), which normalized following glucose infusion therapy. Persistently elevated lactate levels were observed (pyruvate measurement was unavailable at the admitting institution), with a progressive increase throughout hospitalization: 4.1→6.0→7.9→8.0 mmol/L (reference interval: 0.5–1.6 mmol/L). Only partial and transient reductions were achieved with infusion therapy (to 5.0–6.0 mmol/L).

A sustained reduction in base excess (BE) with a fluctuating pattern was concurrently recorded: −7.0; + 0.4; −11.1; −5.4; −5.4; −3.6; −9.1, collectively indicative of persistent metabolic acidosis refractory to treatment.

Upon completion of antiviral therapy, clinical recovery from CMV infection, and resolution of the catarrhal syndrome, the patient was transferred to the cardiology unit for further evaluation and management.

Cardiological assessment revealed symmetrical non-obstructive hypertrophy of the left and right ventricular myocardium and the interventricular septum, with signs of circulatory insufficiency grade IIA.

**FIGURE 2:**
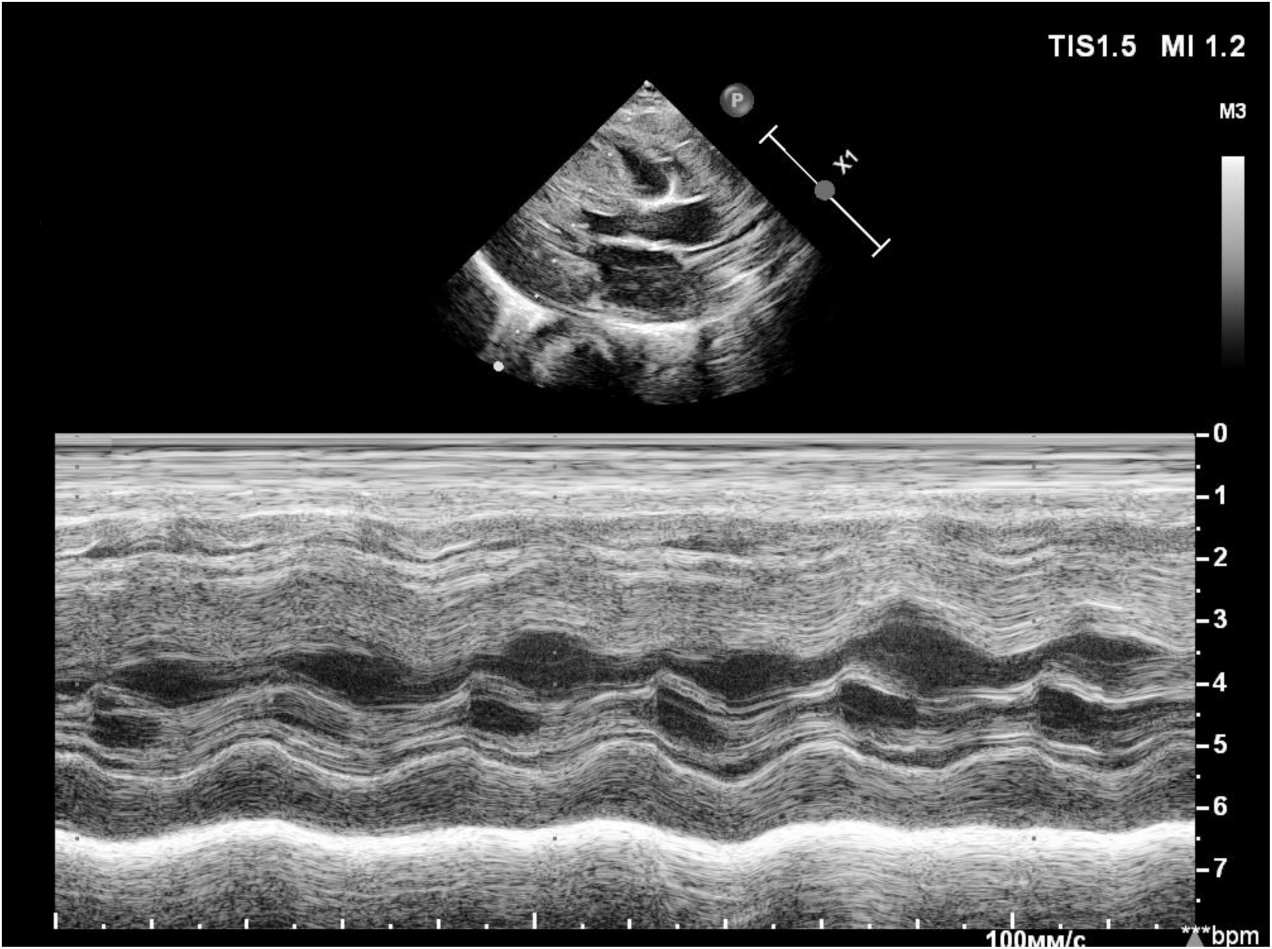
Cardiac ultrasound of the proband.

Echocardiographic findings demonstrated symmetrical hypertrophy: diastolic interventricular septal thickness (IVSd) 9.0 mm (z-score 3.76), diastolic left ventricular posterior wall thickness (LVPWd) 8.3 mm (z-score 4.82). Myocardial mass 60.66 g (z-score 6.7), myocardial mass index (MMI) 10.89 g/kg, MMI/BSA 193.34 g/m^2^. Diastolic right ventricular anterior wall thickness was 4.0 mm (reference: 3–4 mm). No significant left ventricular outflow tract obstruction was found (PGr LVOT 6 mmHg). A patent foramen ovale of 3 mm diameter with left-to-right shunting was identified. Left ventricular cavity dimensions were within normal limits: LV end-diastolic diameter (EDD) 25.0 mm (z-score 1.06), LV end-diastolic volume (EDV) 22.32 mL. Left ventricular systolic function was preserved: left ventricular ejection fraction (LV EF) 70.43%, fractional shortening (FS) 38.0%. Grade I mitral regurgitation and mild dilatation of the left coronary artery (2.2 mm, z-score 2.31) were documented. Inferior vena cava collapsibility exceeded 50% (Figure 2).

**FIGURE 3:**
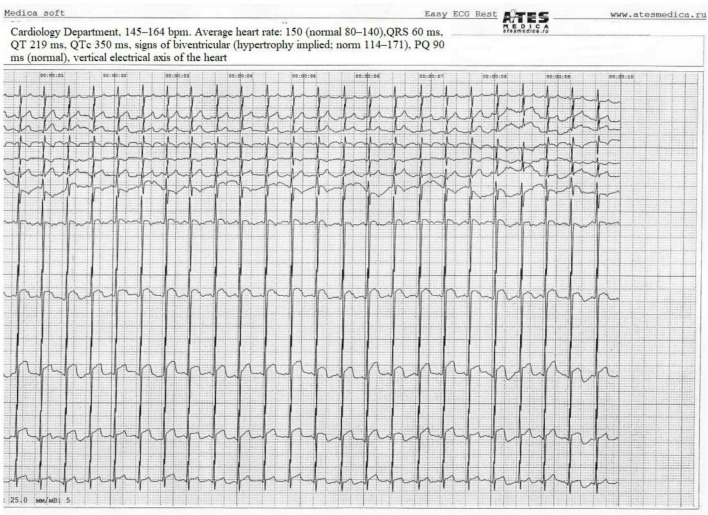
Resting ECG of the proband.

Standard 12-lead ECG demonstrated sinus rhythm with episodes of sinus arrhythmia, signs of biventricular myocardial hypertrophy, and isolated supraventricular and ventricular ectopic beats. Twenty-four-hour Holter monitoring revealed a tendency toward tachycardia: mean heart rate 143 bpm (reference: 131 ± 5 bpm), with predominance of atrial rhythm during nocturnal hours, 5 supraventricular extrasystoles, and 1 ventricular extrasystole (Figure 3).

Troponin I was within the reference range at 0.1 ng/mL (reference: 0–0.16 ng/mL). NT-proBNP was markedly elevated at 3,055 ng/mL (reference: < 200 ng/mL), confirming hemodynamically significant heart failure.

Physical examination revealed minimal and non-specific dysmorphic features (microstomia, enlarged upper alveolar ridge, altered plantar dermatoglyphics), without any clear indication of a syndromic condition. The absence of an orienting response to auditory stimuli was additionally noted, raising suspicion of hearing impairment. In the context of recent CMV infection, otoacoustic emissions were assessed bilaterally: no pathology of the peripheral auditory system was identified; repeat audiological screening was recommended at 1 month.

To exclude lysosomal storage disorders as a cause of myocardial hypertrophy, enzymatic diagnostics were performed by tandem mass spectrometry (TMS) on dried blood spot cards. Activities of galactocerebrosidase (Krabbe disease), alpha-glucosidase (Pompe disease), alpha-galactosidase (Fabry disease), sphingomyelinase (Niemann–Pick disease type A/B), and alpha-iduronidase (mucopolysaccharidosis type I) were all within reference ranges, effectively excluding these diagnoses. Urinary glycosaminoglycan levels (dermatan sulfate, chondroitin sulfate, heparan sulfate, and keratan sulfate), measured by HPLC-MS/MS, were likewise within normal limits.

Urinary organic acid analysis by GC-MS revealed markedly elevated concentrations of several metabolites but 2-methylacetoacetate (Table 3). The laboratory interpretation indicated that the observed abnormalities were non-specific, and can represent ketosis/ketoacidosis or number of inherited metabolic disorders, such as mitochondrial hepatopathies, fumaric aciduria, or 2-methyl-3-hydroxybutyric aciduria, and required careful correlation with clinical findings.

**TABLE 3:** Urinary organic acid profile by GC-MS analysis (mmol/mol creatinine).

Metabolite	Result	Reference range	Fold elevation
Lactate	2450.70	0–25	↑
2-Oxoglutaric acid	906.85	0–152	↑
3-Hydroxybutyrate	629.47	0–3	↑
Fumaric acid	361.06	0–37.7	↑
2-Hydroxyisobutyrate	350.56	0–2	↑
4-Hydroxyphenyllactate	350.25	6–28	↑
4-Hydroxyphenylpyruvate	178.29	0–2	↑
3-Hydroxysebacic acid	128.86	0–2	↑
3-Methylglutaconic acid	51.37	0–9	↑
Adipic acid	28.63	0–12	↑
Glutaric acid	24.77	0–2	↑
2-Methyl-3-hydroxybutyrate	19.60	0–11	↑
Homovanillic acid	19.25	2–15	↑
Ethylmalonic acid	15.91	0–7	↑
Pyruvate	13.28	0–12	↑
Succinate	12.24	0.5–16	Within range
Suberic acid	10.94	0–2	↑
2-Oxoadipic acid	9.23	0–2	↑
Methylsuccinate	4.52	0–3	↑
Vanillyllactate	3.02	0–0.6	↑
Tiglylglycine	2.80	0–2	↑

GC-MS, gas chromatography–mass spectrometry. All values are expressed as mmol/mol creatinine. Values within the reference interval are indicated accordingly. ↑ denotes elevation above the upper reference limit.

Taking into account nonspecific character of the biochemical abnormalities, blood samples were directly referred for molecular genetic diagnostics.

In the light of the suspected inherited metabolic disorder, metabolic-energetic and cardioprotective therapy was initiated: levocarnitine 100 mg/day and spironolactone 12.5 mg/day.

Despite persistent lactic acidosis, no clinical signs of metabolic crisis were observed most likely due to the continuous supportive therapy. The patient was discharged with recommendations: long fasting intervals avoidance, spironolactone 6.25 mg twice daily (2 mg/kg/day), ubidecarenone (coenzyme Q10) 3 drops once daily for 3 months, levocarnitine 300 mg twice daily (100 mg/kg/day) for 3 months, repeat audiological assessment at 1 month; planned admission specialized Pediatric Cardiology Unit in 3 months, and in case of unexplained episodes of weakness or lethargy—immediate admission to intensive care department. Riboflavin therapy was not started, as the patient was discharged before genetic testing results.

Whole-exome sequencing was performed on the Illumina NovaSeq 6000 platform using the SureSelect All Exon V7 capture kit. Variant interpretation was conducted in accordance with the criteria of the American College of Medical Genetics and Genomics (ACMG/AMP). Written informed consent from the proband’s parents for genetic testing, segregation analysis, and publication of anonymized data was obtained on 28 December 2024.

**FIGURE 4:**
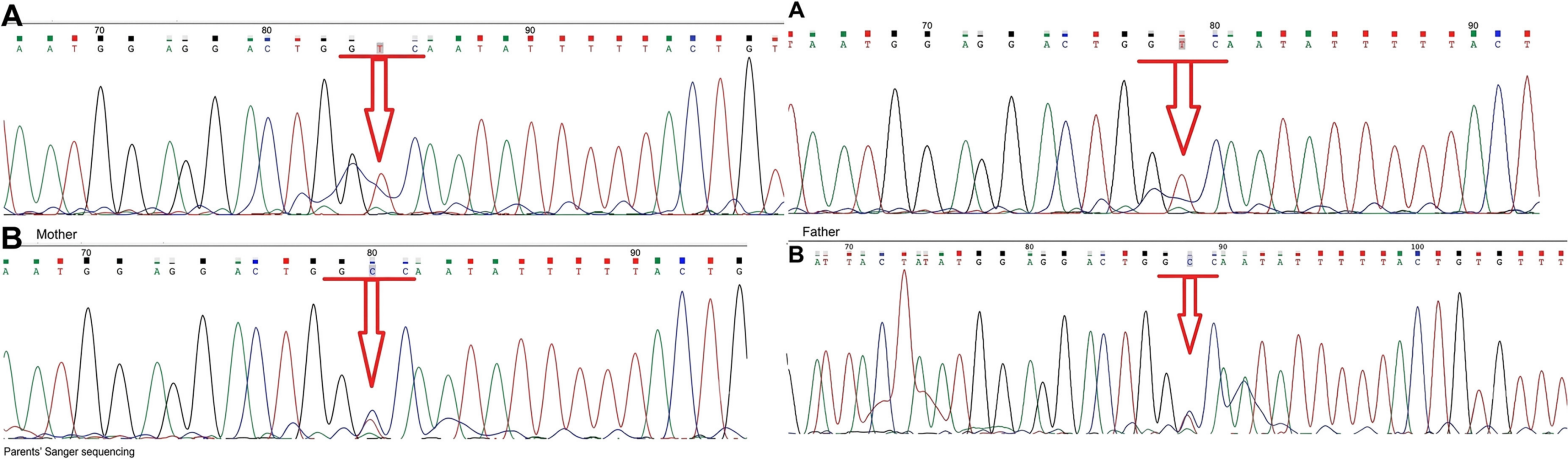
Results of the proband’s **(A)** and family **(B)** genetic testing.

Molecular genetic analysis identified a rare nucleotide variant c.659C > T (p.Ala220Val) in the *ACAD9* gene in the homozygous state. According to the ClinGen clinical validity classification framework, *ACAD9* holds the highest level of evidence (Definitive) for its association with fatty acid oxidation disorders (7). The variant was classified as likely pathogenic (Class IV) per ACMG/AMP criteria (8) and deposited in ClinVar (VCV004526405.1). Carrier status and zygosity were confirmed in the proband and her parents by Sanger sequencing (Figures 4A,B).

It was subsequently learned that the girl died at her place of residence due to a metabolic crisis and acute cardiovascular failure prior to receipt of the genetic testing results.

**FIGURE 5:**
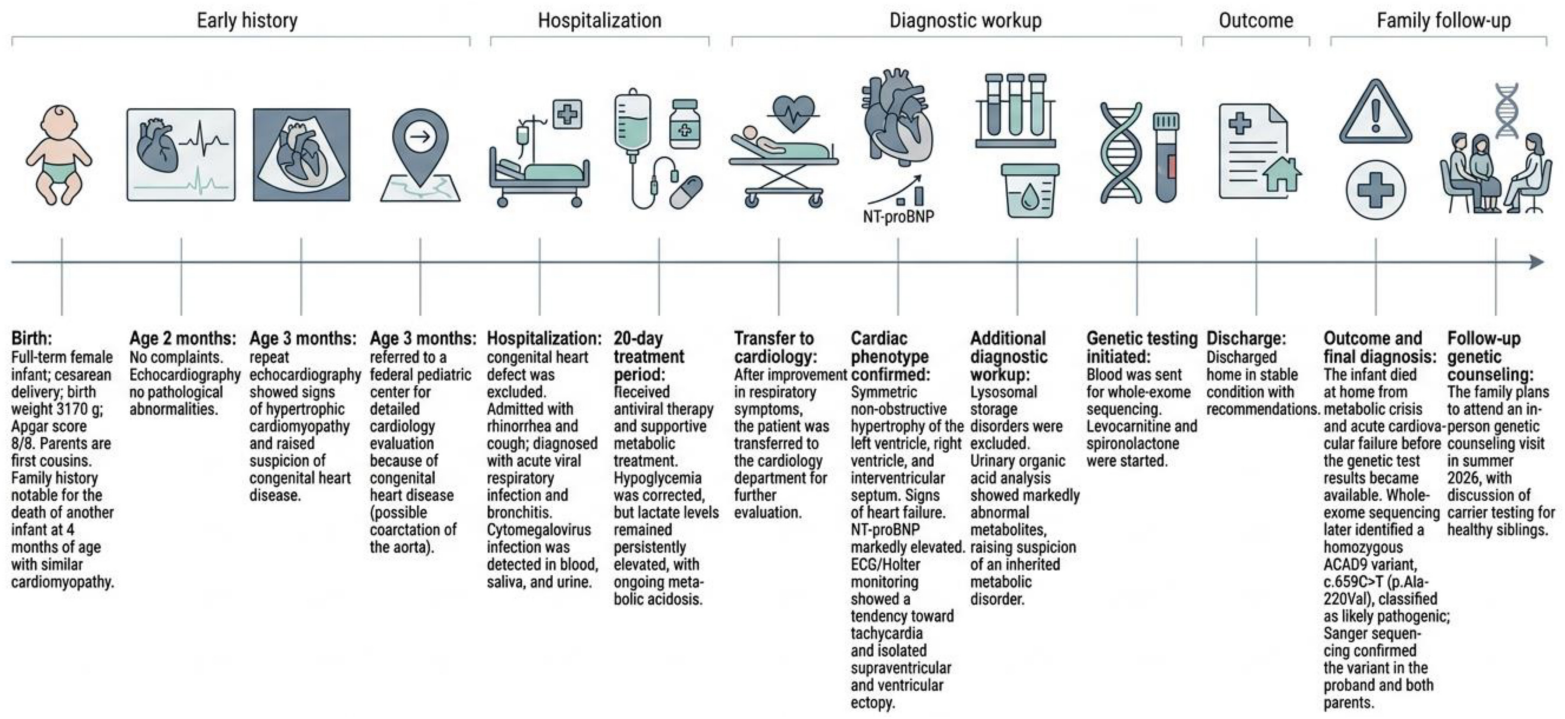
Clinical timeline of an infant with hypertrophic cardiomyopathy and subsequent diagnosis of ACAD9 deficiency.

Medical genetic counseling was offered to the family based on the molecular diagnostic findings, along with carrier testing for the identified variant in healthy siblings. The family expressed interest in the consultation; however, given the considerable distance from the center (over 1,500 km), an in-person appointment is most likely to be arranged during the summer of 2026. The key milestones of the clinical history are presented in Figure 5.

## 3 Discussion

This case illustrates the severe early-onset course of metabolic LVH in a child of a consanguineous family. The clinical onset of myocardial hypertrophy, hypotonia, and metabolic acidosis in the first months of life is consistent with the reported spectrum of ACAD9 deficiency, which is frequently associated with isolated mitochondrial respiratory chain complex I deficiency and early HCM (3–5, 9).

ACAD9 was originally characterized as a member of the acyl-CoA dehydrogenase family with activity toward long-chain unsaturated acyl-CoA substrates (10). Subsequent work, however, established that its dominant physiological role in most tissues is not in fatty acid β-oxidation but in the assembly of mitochondrial respiratory chain complex I, where ACAD9 interacts with the assembly factors NDUFAF1 and ECSIT as a component of the mitochondrial complex I assembly complex (11, 12). This explains why biallelic pathogenic variants in ACAD9 typically manifest as isolated complex I deficiency rather than as a classical fatty acid oxidation disorder, and why serum acylcarnitine profiles are often unremarkable in affected patients (5, 11, 13). In the proband, normal acylcarnitine analysis combined with persistent lactic acidosis and hypertrophic cardiomyopathy is therefore in keeping with a primary defect in complex I biogenesis.

Its pathophysiology is driven by impaired mitochondrial energy metabolism, resulting in insufficient adenosine triphosphate (ATP) production, particularly during increased metabolic demands or limited substrate availability. Energy deficiency promotes a shift toward anaerobic glycolysis with lactate accumulation and contributes to compensatory myocardial hypertrophy. Energy failure and metabolic acidosis may injure cardiomyocytes and manifest clinically as progressive heart failure (12). The long chain fatty acids are a major oxidative substrate for the myocardium, and disruption of β-oxidation may further restrict ATP resynthesis and aggravate the phenotype (3, 14). ACAD9 is also the predominant long-chain acyl-CoA dehydrogenase in the central nervous system (10), which may contribute to the muscular hypotonia and neurological features observed in severely affected infants. Accordingly, persistent lactic acidosis and hypotonia in the proband aligned with the typical multisystem presentation of ACAD9 deficiency (3, 4).

The ACAD9 c.659C > T (p.Ala220Val) variant identified in the patient has a compelling functional relevance. The Ala220Val substitution is located near the catalytic core of ACAD9 and has been shown to disrupt protein folding and stability, resulting in severely reduced ACAD9 and complex I protein levels in patient fibroblasts, with only partial rescue upon lentiviral complementation (9). Structural and functional studies of 16 ACAD9 missense variants have further demonstrated that mutations affecting the catalytic portion of the protein—as is the case for Ala220Val—tend to abolish both dehydrogenase activity and complex I assembly, whereas variants localized to the C-terminal domain more often preserve catalytic activity (15). The inverse correlation between residual enzyme activity and clinical severity reported in that study is consistent with the severe neonatal presentation in this observation.

Independent observations further strengthened evidence supporting the pathogenicity of this variant. A similar clinical phenotype has been reported in the context of parental consanguinity, and riboflavin supplementation did not ameliorate complex I deficiency in patient fibroblasts, suggesting a riboflavin-nonresponsive disease course for this genotype (9).

The phenotypic spectrum of ACAD9 deficiency, however, is broad. Neonatal and even prenatal presentations with intrauterine growth restriction, fetal cardiomegaly and lethal lactic acidosis shortly after birth have been described (4, 16, 17), while other patients present in later childhood or adolescence with isolated cardiac hypertrophy, exercise intolerance or even atypical neurological features such as microcephaly, dystonia and Leigh-like phenotypes without overt cardiomyopathy (13, 18). A series of nine patients from three unrelated families demonstrated that cardiac involvement may range from electrical left ventricular hypertrophy and mild echocardiographic hypertrophy to severe hypertrophic, dilated or combined cardiomyopathy, and that patent ductus arteriosus may occur more frequently than expected by chance (13). This intrafamilial and interfamilial variability indicates that the phenotype cannot be reliably predicted from genotype alone.

Importantly, the response to riboflavin therapy in patients with ACAD9 deficiency is heterogeneous. The original description of riboflavin-responsive complex I deficiency due to a homozygous p.Arg532Trp variant established that flavin supplementation can improve exercise tolerance and complex I activity in selected patients (19). In the largest cohort reported to date (70 patients), riboflavin increased complex I activity in 9 of the 15 analyzed cell lines, whereas no effect was observed in the remaining cases (3). Early infantile onset in riboflavin-nonresponsive genotypes is generally associated with an unfavorable prognosis (3). Early initiation of riboflavin in responsive patients, on the other hand, may substantially alter the natural history of the disease: long-term survival into adulthood and even an uneventful pregnancy have been reported (20).

Additional clinical case with the same homozygous mutation genotype has been reported, in which the child remained alive at 18 months of age at the time of publication while receiving combined metabolic therapy (21). In such cases, stabilization may reflect comprehensive metabolic support rather than a specific riboflavin response. For riboflavin-nonresponsive patients, several alternative pharmacological strategies have been explored. Combination therapy with sodium pyruvate, β-blocker and coenzyme Q10 produced sustained normalization of left ventricular function in an infant with progressive ACAD9-related cardiomyopathy who had not received riboflavin (22). Bezafibrate combined with nicotinamide riboside has been reported to transiently stabilize cardiomyopathy and reduce lactate and NT-proBNP levels in a riboflavin-unresponsive infant, although long-term outcome remained fatal (23). These observations underscore that no uniformly effective disease-modifying therapy is currently available, and therapeutic decisions must be individualized based on genotype, riboflavin responsiveness and organ involvement.

In early childhood HCM is often first considered within the spectrum of sarcomeric diseases, for which medium-term survival is typically higher than that of mitochondrial and metabolic cardiomyopathies (1, 24).

In the present family, parental consanguinity, early death of a sibling with a similar presentation, and systemic features suggestive of metabolic involvement (lactic acidosis, hypotonia, and hepatosplenomegaly) shifted the initial diagnostic suspicion toward an inherited metabolic disorder. This combination of findings should be considered as an indication to the prompting early molecular genetic testing.

The diagnostic strategy included WES, followed by Sanger validation of the detected variant in the proband and her parents. This approach is consistent with current recommendations for children with unexplained cardiomyopathy and suspected mitochondrial disease, particularly when the phenotype is multisystemic and targeted gene panels may not cover relevant genes (25, 26). Because ACAD9 deficiency typically presents with normal or only mildly abnormal biochemical markers and cannot be reliably diagnosed by standard metabolic screening, molecular testing has become the primary diagnostic tool (11, 18). In rapidly progressive neonatal cases with a lethal outcome, post-mortem molecular analysis combined with histopathological evaluation - including immunohistochemical demonstration of mitochondrial hyperplasia in the heart, liver, skeletal muscle and renal tubules - has been shown to establish the diagnosis and inform reproductive counseling for the family (17).

The practical value of this case is not limited to refining the phenotypic spectrum of ACAD9 deficiency but also highlights the role of molecular diagnosis for family and reproductive counseling. Establishing autosomal recessive inheritance and confirming a specific pathogenic variant allows parents to be informed about the risk of recurrence (25% for any pregnancy) and consider prenatal diagnosis or preimplantation genetic testing for monogenic disease (PGT-M).

WES is commonly used to identify the molecular cause of suspected ACAD9 deficiency. Standard WES turnaround times typically range from 2 to 8 weeks or longer and may increase further when multiple sequential genetic tests are required (27). For critically ill infants, such timelines can be clinically unacceptable, because the disease may progress rapidly and require timely therapeutic decisions. Heart transplantation may be considered as a last-resort option for life-threatening cardiomyopathy; however, its effectiveness in ACAD9 deficiency is constrained by the systemic nature of mitochondrial defects. Delayed neurological and muscular manifestations (cognitive impairment, seizures, weakness, exercise intolerance), renal tubular involvement, and optic atrophy have been reported (6). These data suggest that isolated correction of cardiac pathology does not address the underlying metabolic defects and may not prevent the progression of multisystem diseases, necessitating a careful, individualized approach to transplant decision-making.

Given the unfavorable prognosis of severe infantile-onset disease and the autosomal recessive inheritance pattern conferring a 25% risk of recurrence, families with a confirmed molecular diagnosis should receive genetic counseling with a detailed discussion of reproductive options. When both parents are confirmed carriers of pathogenic variants, prenatal molecular testing and PGT-M can be used to reduce the risk to the affected child in future pregnancies.

## 4 Conclusion

This case highlights the severe and rapidly progressive course of infantile HCM caused by a homozygous pathogenic variant in the ACAD9 gene. Early myocardial hypertrophy accompanied by lactic acidosis, hypotonia, hepatosplenomegaly, and positive family history in a consanguineous family should raise strong suspicion for a metabolic cardiomyopathy rather than a primary sarcomeric disorder. Although molecular diagnosis may not alter the outcome in fulminant neonatal forms, it is essential for accurate prognosis, optimization of metabolic management, and informed genetic counseling. Identification of autosomal recessive inheritance enables precise recurrence risk assessment and consideration of prenatal or preimplantation genetic testing in future pregnancies. Early integration of rapid genomic testing into the evaluation of infants with unexplained cardiomyopathy is particularly important in regions with a high prevalence of consanguinity marriages.

## Data Availability

The data presented in the study are deposited in the ClinVar repository (https://www.ncbi.nlm.nih.gov/clinvar/), accession number VCV004526405.1.
